# Deep learning for Chilean native flora classification: a comparative analysis

**DOI:** 10.3389/fpls.2023.1211490

**Published:** 2023-09-11

**Authors:** Carola Figueroa-Flores, Pablo San-Martin

**Affiliations:** ^1^Department of Computer Science and Information Technology, Universidad del Bío Bío, Chillán, Chile; ^2^School of Computer and Information Engineering, Universidad del Bío-Bío, Chillán, Chile

**Keywords:** image classification, Chilean native flora, convolutional neural network, deep learning, transfer learning

## Abstract

The limited availability of information on Chilean native flora has resulted in a lack of knowledge among the general public, and the classification of these plants poses challenges without extensive expertise. This study evaluates the performance of several Deep Learning (DL) models, namely InceptionV3, VGG19, ResNet152, and MobileNetV2, in classifying images representing Chilean native flora. The models are pre-trained on Imagenet. A dataset containing 500 images for each of the 10 classes of native flowers in Chile was curated, resulting in a total of 5000 images. The DL models were applied to this dataset, and their performance was compared based on accuracy and other relevant metrics. The findings highlight the potential of DL models to accurately classify images of Chilean native flora. The results contribute to enhancing the understanding of these plant species and fostering awareness among the general public. Further improvements and applications of DL in ecology and biodiversity research are discussed.

## 1 Introduction

Chile’s expansive geographical territory encompasses a wide array of flora, influenced by its diverse climatic conditions. However, information regarding native flora is often restricted to informative panels with minimal details, primarily found in specific physical locations within national parks and protected areas (Rodriguez et al., 2018). This limited availability of information hinders visitors from acquiring comprehensive knowledge about the country’s native flora, resulting in a low societal appreciation of wildlife and an insufficient understanding of the significance of biodiversity conservation. To address this issue, it is crucial to comprehend people’s attitudes and intentions towards wildlife and explore the relationships between humans and various species within local ecosystems. In this context, technological advancements, such as computers, the internet, television, and video games, are considered to have contributed to a decrease in personal experiences with nature, consequently diminishing sensitivity towards environmental issues. In order to effectively conserve biodiversity, it is crucial to consider the perceptions of individuals towards their natural environment. Proposed models of environmental perception should acknowledge humans as information processors and organizers, capable of constructing a coherent representation of the world to address challenges. One approach that aligns with this perspective is the utilization of computational image classification methods, which employ Deep Learning (DL) techniques to accurately recognize and classify species depicted in images. DL is widely recognized for its exceptional performance in solving real-world problems, as well as its capacity to handle large volumes of data. The ease with which relevant features can be extracted during the learning process, coupled with the utilization of Graphic Processing Units (GPUs), further expedites the learning process. DL is extensively employed by companies to extract knowledge from data generated by electronic devices, ranging from computers to smartwatches or activity-tracking bracelets. By leveraging DL, valuable insights regarding native flora can be obtained, ultimately playing a pivotal role in the conservation of Chile’s biodiversity (Rodriguez et al., 2018; Carranza et al., 2020).

Image classification algorithms can be classified into three main categories: supervised, unsupervised, and weakly supervised. In supervised classification, the user selects a representative sample of pixels from an image to train the algorithm. Unsupervised classification, on the other hand, groups pixels based on common characteristics without the need for userdefined sample classes. Weakly supervised classification algorithms utilize weaker forms of supervision and can employ complete, exact, or inexact supervision.

There are several well-known classification algorithms used in image analysis. Convolutional Neural Networks (CNNs) have gained significant attention due to their outstanding performance in image classification tasks. Artificial Neural Networks (ANNs) are also widely used, as they mimic the structure and function of the human brain. Support Vector Machines (SVMs) excel in both classification and regression tasks, aiming to find an optimal hyperplane to separate different classes. K-Nearest Neighbors (KNN) is a straightforward yet powerful algorithm that assigns a class label to a new data point based on the labels of its nearest neighbors. Na¨ıve Bayes classifiers rely on Bayes’ theorem and assume independence among features given the class label. Finally, Random Forest is an ensemble learning method that combines multiple decision trees for making predictions.

These algorithms offer diverse approaches to image classification, each with its own strengths and weaknesses. The choice of algorithm depends on the specific task requirements, dataset characteristics, and desired performance. Researchers and practitioners should carefully evaluate and select the most suitable algorithm for their specific application to achieve accurate and reliable classification results (Sen et al., 2020).

When it comes to flora classification, deep learning using CNN algorithms emerges as the most effective method. Since 2012, CNNs have established themselves as the primary algorithm for image classification. They have demonstrated exceptional accuracy in various visual recognition tasks such as object detection, localization, and semantic segmentation.

To identify the most suitable CNN for classifying native Chilean flora, a comparative analysis of renowned CNNs will be conducted. This analysis will include Inception, VGG19, MobileNet, and ResNet 152, all of which will be pre-trained on ImageNet. The evaluation will be performed on a comprehensive dataset consisting of over 5000 images of native Chilean flora, incorporating transfer learning techniques to leverage pre-existing knowledge from the ImageNet dataset. By examining and comparing the performance of these CNN models, we aim to determine the optimal choice for accurate and efficient classification of native Chilean flora.

We briefly summarize below our main contributions:

- We create a dataset of Chilean native flora species by labeling and augmenting images to enhance the model’s training,
- We conducted a comparative analysis of the most wellknown CNN models to determine which one provided better accuracy in the classification task,
- After conducting experiments and evaluations, we determined which CNN model delivers better results in the task of classifying native Chilean flora with an accuracy rate of 90% with transfer learning.

The paper is organized as follows. Section II provides a comprehensive review of the related work in image classification, with a specific focus on the classification of Chilean native flora. This section examines the existing literature, highlighting key studies and approaches in the field.

In Section III, we present our proposed approach for classifying native Chilean flora using CNN models. We discuss the selection and configuration of the CNN models, as well as the preprocessing steps and training procedures employed in our methodology.

Section IV presents the experimental results of our study. We provide a detailed analysis of the performance of the CNN models on the native Chilean flora dataset, including accuracy, precision, recall, sensitivity, specificity, F-Score, AUC and time in milliseconds. Additionally, we discuss any notable findings or insights obtained from the experiments.

Finally, in Section V, we draw our conclusions based on the outcomes of our research. We summarize the main findings, discuss their implications for the classification of native Chilean flora, and highlight potential avenues for future research and development in this field.

By following this organization, we aim to provide a clear and structured presentation of our study, allowing readers to easily navigate and comprehend the content of the paper.

## 2 Related work

### 2.1 Convolutional neural networks

Convolutional Neural Networks (CNNs) have garnered significant attention and have been extensively explored in diverse domains, including computer vision, natural language processing, and speech recognition. Within the realm of computer vision, CNNs have demonstrated remarkable accomplishments in a wide range of tasks, encompassing image classification, object detection, semantic segmentation, and image generation (Sarigul et al., 2019; Zhou, 2020). Their inherent ability to effectively capture and extract meaningful features from images has contributed to their widespread adoption and success in various visual recognition tasks. The utilization of CNNs has propelled advancements in the field of computer vision, paving the way for enhanced capabilities and improved performance in tasks that require sophisticated understanding and interpretation of visual data (Maggiori et al., 2017; Ashraf et al., 2019; Hui et al., 2020; Chen et al., 2021; Zelenina et al., 2022).

In recent years, there has been an escalating interest in enhancing the performance and efficiency of Convolutional Neural Networks (CNNs). This has led to significant advancements in the field, with notable contributions encompassing the development of novel architectural designs, including ResNet, Inception, MobileNet, and VGG. These architectures have been specifically engineered to reduce the number of parameters while simultaneously preserving or even improving classification accuracy. In addition to architectural innovations, other approaches have been explored to optimize the training process of CNNs. These include leveraging transfer learning, data augmentation, and regularization techniques. Transfer learning enables the utilization of pre-trained models on largescale datasets to improve generalization and efficiency. Data augmentation techniques enhance model robustness by artificially expanding the training dataset through various transformations and perturbations. Regularization techniques, on the other hand, impose constraints on the model’s parameters to mitigate overfitting and enhance generalization. Collectively, these research efforts aim to refine and optimize CNNs, leading to improved performance and more efficient utilization in various computer vision tasks (Zhu and Chang, 2019; Pattnaik et al., 2021; Ruchai et al., 2021; Wang and Lee, 2021; Bahmei et al., 2022; She et al., 2022).

CNNs have found wide-ranging applications in specific domains, including medical imaging, wherein they have demonstrated promising outcomes in critical tasks such as disease diagnosis, tumor detection, and lesion segmentation. Their ability to extract intricate visual features has enabled significant advancements in the field of medical diagnostics. Moreover, CNNs have made substantial contributions to areas like robotics, autonomous driving, and other domains that necessitate real-time visual processing. By leveraging CNNs, researchers and practitioners have been able to enhance the perception and decision-making capabilities of intelligent systems, enabling them to operate effectively and autonomously in dynamic environments. The utilization of CNNs in these domains showcases their versatility and efficacy in addressing complex visual challenges and underscores their potential to revolutionize various fields reliant on real-time visual analysis (Pham and Jeon, 2017; Aoki et al., 2018; Kocic et al., 2019; Lim et al., 2020; Lozhkin et al., 2021; Li et al., 2022; Roostaiyan et al., 2022).

Overall, Convolutional Neural Networks (CNNs) have emerged as a fundamental tool in the field of computer vision and remain an active area of ongoing research and development. Their exceptional performance in various visual tasks has solidified their importance and relevance.

In the context of this study, our objective is to conduct a comprehensive analysis of the four most renowned models utilized for image classification, particularly focusing on their applicability in classifying images of native Chilean flora. In the subsequent sections, we will provide detailed explanations and insights into these selected models.

#### 2.1.1 ResNet152

ResNet152 is a convolutional neural network architecture with deep layers that was developed by researchers at Microsoft in 2015. The architecture introduces a novel concept called residual learning, wherein the network is trained to learn residual functions instead of directly mapping the input to the output. This innovative approach enables the network to become significantly deeper than previous architectures, while still achieving remarkable performance (He et al., 2016).

ResNet152 is a convolutional neural network architecture that consists of 152 layers. It utilizes skip connections, also known as residual connections, to facilitate the flow of gradients during backpropagation. These skip connections allow the network to simultaneously learn both low-level and highlevel features, making it highly effective for image recognition tasks.

Additionally, ResNet152 incorporates batch normalization, a technique that helps mitigate overfitting and accelerates the training process. By normalizing the activations within each batch, batch normalization enhances the network’s stability and enables more efficient learning.

ResNet152 has demonstrated outstanding performance on prominent image recognition benchmarks, including ImageNet, which comprises over a million images across 1,000 classes. Its exceptional results have made it a state-of-the-art model in the field. Moreover, ResNet152 has found applications in diverse domains, such as object detection, image segmentation, and face recognition, showcasing its versatility and effectiveness across multiple tasks.

#### 2.1.2 VGG19

VGG19 is a convolutional neural network (CNN) model proposed by the Visual Geometry Group (VGG) at the University of Oxford in 2014. It is characterized by its architecture, consisting of 19 layers comprising convolutional and pooling layers, followed by three fully connected layers.

One of the notable aspects of VGG19 is its extensive use of small 3x3 convolutional filters across the network. This design choice allows the model to effectively capture local features and their combinations, enhancing its ability to generalize well to new images. Moreover, the depth of the VGG19 architecture enables it to learn increasingly complex features as the network goes deeper.

VGG19 is commonly employed as a pre-trained model for transfer learning in various computer vision tasks. Pretrained models, such as VGG19, have already learned weights from large-scale datasets like ImageNet. Leveraging these pretrained weights as a starting point can greatly benefit new tasks that involve smaller datasets, as it helps accelerate the learning process and improve performance.

By utilizing VGG19 as a pre-trained model, researchers and practitioners can leverage the knowledge and representations learned from vast image datasets, enabling them to tackle new visual recognition problems more effectively (Simonyan and Zisserman, 2014).

#### 2.1.3 InceptionV3

InceptionV3 is a convolutional neural network (CNN) architecture that was introduced in 2015 by researchers at Google. It is a deep neural network with 48 layers, and it was designed specifically for image recognition and classification tasks.

InceptionV3 uses a unique module called an “Inception module” that is able to perform multiple convolutions and pooling operations at different scales in parallel. This allows the network to capture both local and global features in the image, making it more accurate at recognizing complex patterns.

The network was trained on the ImageNet dataset, which is a large dataset of over 14 million images. During training, the network learned to classify images into one of 1,000 different categories, such as “dog”, “cat”, or “car”.

InceptionV3 has been used in many applications, including object recognition, facial recognition, and medical image analysis. Its high accuracy and ability to handle complex images make it a popular choice for deep learning practitioners (Szegedy et al., 2015).

#### 2.1.4 MobileNetV2

MobileNetV2 is an architecture of convolutional neural network (CNN) introduced in 2018 as an enhancement to the original MobileNet model. It addresses the need for a lightweight network that can deliver high accuracy in image classification tasks while minimizing computational requirements.

The primary concept behind MobileNetV2 involves utilizing a combination of depthwise separable convolutions and linear bottlenecks. Depthwise separable convolutions involve breaking down the standard convolution into two distinct layers: a depthwise convolution and a pointwise convolution. The depthwise convolution applies individual filters to each input channel, while the pointwise convolution merges the outputs from the depthwise convolution through a linear transformation. By doing so, the computational complexity of the convolution operation is reduced while preserving accuracy.

MobileNetV2 also incorporates linear bottlenecks as a significant element. These bottlenecks serve to diminish the dimensionality of the feature maps while retaining the maximum amount of information. They accomplish this by applying a linear transformation to the feature maps, followed by passing them through an activation function.

Overall, MobileNetV2 stands as an extremely efficient and precise CNN architecture, ideally suited for scenarios with limited resources such as mobile devices and embedded systems. Its utilization of depthwise separable convolutions and linear bottlenecks enables it to strike a balance between computational efficiency and accuracy, making it a valuable choice in resource-constrained environments (Sandler et al., 2018).

### 2.2 Comparison convolutional neural network architecture

InceptionV3, ResNet152, VGG19, and MobileNetV2 are all popular convolutional neural network (CNN) models used in image classification tasks.

InceptionV3 was introduced by Google in 2015 and is a deep CNN with 48 layers. It uses a unique architecture of Inception modules, which are multi-branch convolutional blocks that allow the network to learn both spatial features and channel-wise correlations at different scales. InceptionV3 is known for its high accuracy and is often used in complex image recognition tasks.

ResNet152 is a residual network introduced by Microsoft in 2016. It is a very deep CNN model with 152 layers that uses residual connections to address the problem of vanishing gradients, which can occur in very deep networks. These connections allow the gradient to flow through the network more easily, which improves training and accuracy. ResNet152 has achieved state-of-the-art performance in many image recognition tasks.

VGG19 is a CNN model introduced by the Visual Geometry Group (VGG) at the University of Oxford in 2014. It has 19 layers and uses a simple architecture of repeated convolutional layers followed by max pooling and fully connected layers. VGG19 is known for its simplicity and ease of implementation, and it has achieved high accuracy in many image recognition tasks.

MobileNetV2 is a CNN model introduced by Google in 2018. It is designed for mobile and embedded devices and has a small footprint and low computational cost. It uses depthwise separable convolutions to reduce the number of parameters and computational complexity while maintaining high accuracy. MobileNetV2 is often used in real-time image recognition applications on mobile devices.

When comparing these four CNN models, it is important to consider the specific requirements of the image classification task at hand. In general, InceptionV3 and ResNet152 are more suitable for complex and high-accuracy tasks, while VGG19 and MobileNetV2 are more suitable for simpler tasks with less computational resources available.

### 2.3 Classification models for flora images

The classification of flora images has experienced notable advancements in recent years, primarily due to the progress made in deep learning techniques, notably convolutional neural networks (CNNs) (Lopez-Jimenez et al., 2019; Ibrahim et al., 2022). State-of-the-art models for flora image classification often employ pre-trained CNNs, which demonstrate the ability to accurately recognize intricate image patterns. Furthermore, transfer learning, which involves finetuning pre-trained CNNs using new datasets, has proven to be an effective approach in enhancing the accuracy of flora image classification.

Several widely used CNN models have been successfully employed in flora image classification, including InceptionV3, ResNet152, VGG19, and MobileNetV2. Recent research studies have demonstrated the high accuracy rates achieved by these models in the classification of flora images (Kattenborn et al., 2020). For instance, a study focusing on the classification of Brazilian flora images using deep learning models reported classification accuracies reaching up to 93% for InceptionV3 and ResNet152 (Figueroa-Mata et al., 2022).

In order to overcome the challenges arising from limited labeled data in flora image classification, researchers have explored various techniques, including weakly-supervised and semi-supervised learning methods (Heredia, 2017). Moreover, recent studies have concentrated on enhancing accuracy by incorporating additional data sources, such as spectral and hyperspectral information, and leveraging more advanced CNN architectures (Lazarescu et al., 2004; Singh et al., 2009; Wang et al., 2009).

Overall, the state-of-the-art of classification models for flora images is constantly evolving, and we anticipate further advancements in the near future. The selection of an appropriate model may vary depending on the unique characteristics of the dataset and the specific requirements of the classification task at hand (Sulc and Matas, 2017; Hasan et al., 2020; Ball, 2021; Filgueiras, 2022).

## 3 Proposed method

The objective of this paper is to conduct a comparative analysis of various deep learning (DL) models for the classification of native Chilean flora images. The models under evaluation encompass InceptionV3, VGG19, ResNet152, and MobileNetV2, all of which have been pre-trained using the Imagenet dataset.

### 3.1 Data collection

**Figure 1:**
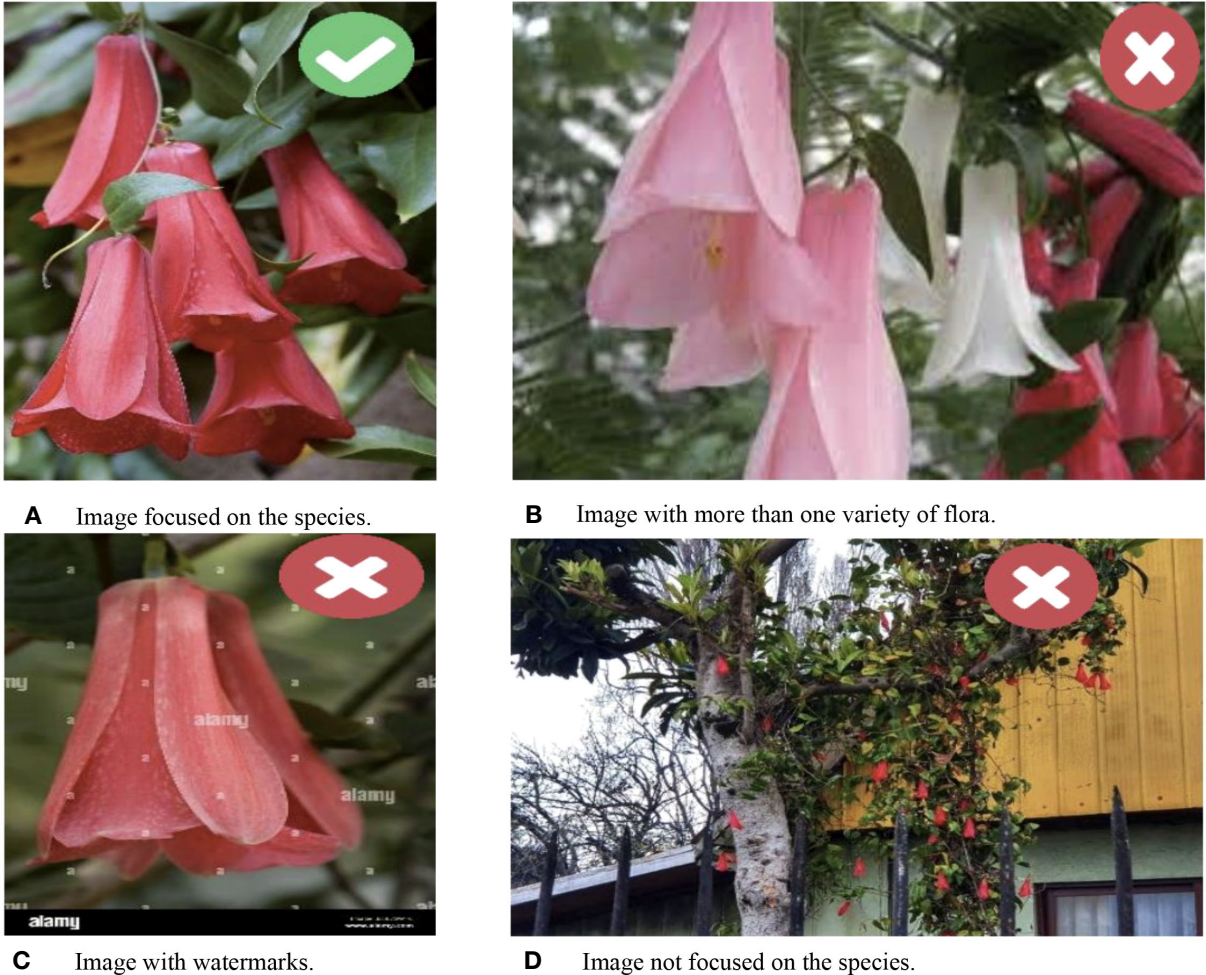
Some samples of chosen and rejected images.

In the process of data collection, the primary source of images was the internet, with careful consideration given to certain limitations and requirements to ensure the preservation of the classes and to obtain a high-quality dataset. To ensure accuracy and avoid interference from external factors such as other flowers, trees, animals, or plants, images were selected based on specific criteria. These criteria included minimal noise, a predominant focus on native species, and the absence of elements that could hinder species identification (refer to **Figure 1**). Furthermore, only real photographs were included, while illustrations of the species were intentionally excluded. Images that contained watermarks or copyright protection preventing their usage, even for non-commercial purposes, were also excluded from the dataset.

The creation of the dataset primarily relied on specialized websites dedicated to documenting flora, such as iNaturalist, Fundacion´ RA Philippi, and Chilebosque. These sources were selected for their comprehensive coverage of native Chilean flora. Additionally, websites focused on general photography, including Pinterest, Flickr, and Alamy, were utilized as secondary sources to enhance the dataset. In cases where the initial results from specialized websites were insufficient in terms of capturing the required flora species, the dataset was augmented with the best results obtained from Google Images while ensuring compliance with the aforementioned specifications and criteria.

The primary objective of this study is to compare the performance of different convolutional neural network (CNN) models, with a focus on analyzing their classification capabilities for Chilean native flora. As such, the resolution of each image used in the study did not significantly impact the CNN models’ classification task. This can be attributed to the following reasons: (i) *Focus on model comparison*: The main objective of this study is to compare the performance of different convolutional neural network (CNN) models. The focus is on analyzing how these models perform in classifying Chilean native flora, regardless of the resolution of the images used. Therefore, the resolution of the images is not a critical factor for evaluating the models’ ability to perform classification. (ii) *Adaptation of images to specific input values*: Regardless of the resolution of each image, all images need to be adapted to the specific input value required by each CNN model. This means that all images are resized to the desired input size, ensuring that all images are processed uniformly, regardless of their original resolution. (iii) *Variability in image quality on the web*: Images collected from different sources on the internet can have a wide range of resolutions and visual quality. Setting a specific resolution standard for all collected images would be impractical and could restrict the diversity and representativeness of the image sample used in the study.

Accordingly, all images are resized to the required input size, and the focus is on evaluating the models’ ability to perform classification effectively. The details of the selected images are in the **Table 1**.

**Table 1:** The details of the selected images.

Website	# Selected Images	# Discarded	Total Images
iNaturalist	1000	320	1320
RA Philippi	500	250	750
Chilebosque	500	250	750
Pinterest	1500	850	2350
Flickr	1000	320	1320
Alamy	500	150	650
Total Images	5000	2140	7000

### 3.2 Native Chilean flora species selected

The chosen flora species are geographically distributed across the country, representing at least one species for each of the main regions (North, Central, and South). Although there are several variations within each species, the selected classes primarily consist of widely recognized varieties, excluding those with minor differences in color tones of the petals or other negligible variations (e.g., number of petals or small spots). Additional details regarding the selected images can be found in the provided **Table 2**.

**Table 2:** Detail our dataset of mages of various species of native Chilean flora.

# Clase	Specie Selected	# Total Images	Region
1	Copihue (Lapageria Rosea)	500	South
2	Chilco (Fuchsia magellanica)	500	South
3	Ana˜ nuca˜ de Fuego (Phycella cyrtanthoides)	500	Central
4	Azulillo (Pasithea caerulea)	500	North
5	Chagual (Puya alpestris)	500	Central
6	Maqui (Aristotelia chilensis)	500	Central/South
7	Lingue (Persea lingue)	500	Central/South
8	Canelo (Drimys winteri)	500	North
9	Quila (Chusquea quila)	500	South
10	Notro (Embothrium coccineum)	500	Central

**Figure 2:**
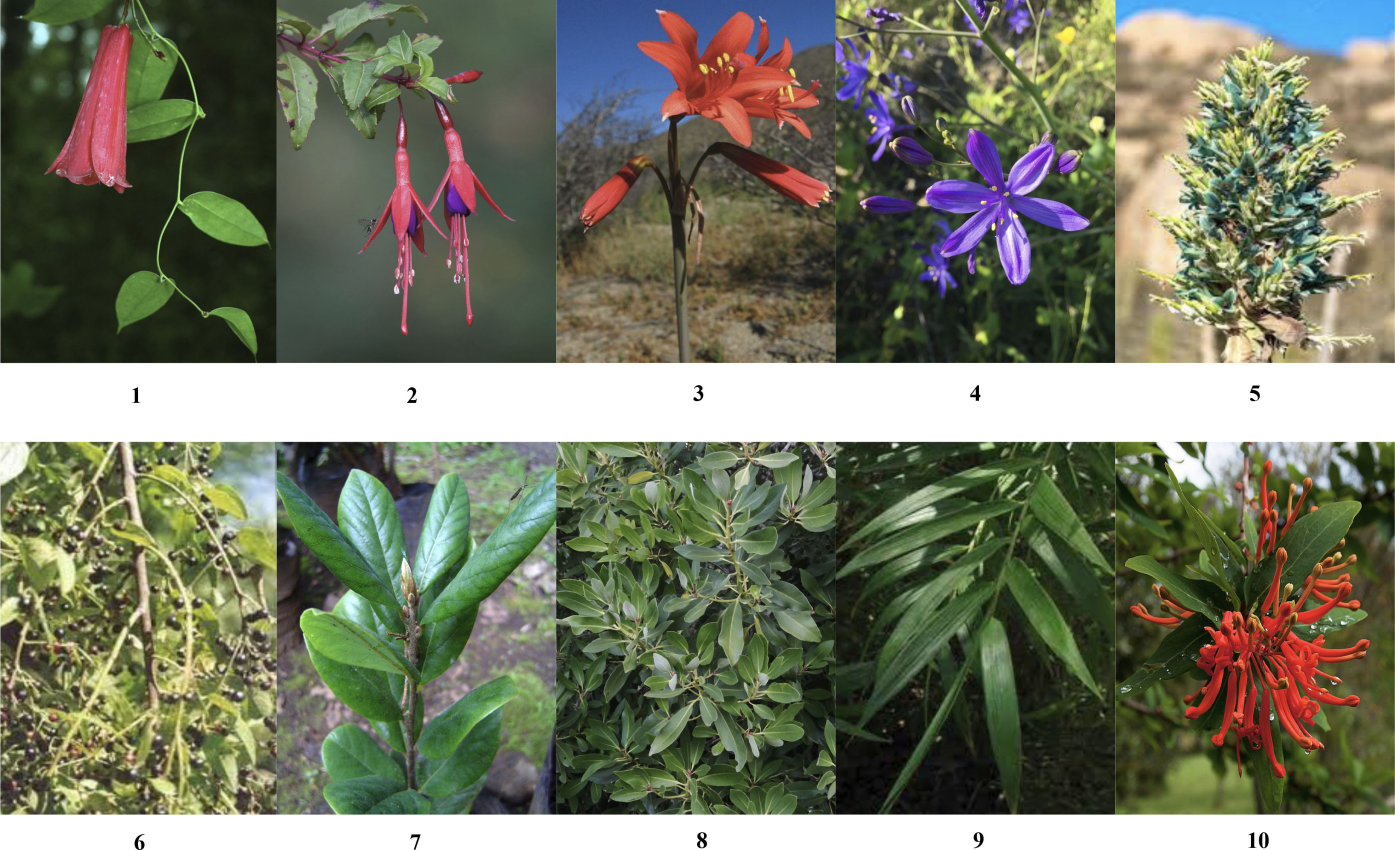
Sample of images depicting ten distinct species of Chilean native flora.

**Figure 3:**
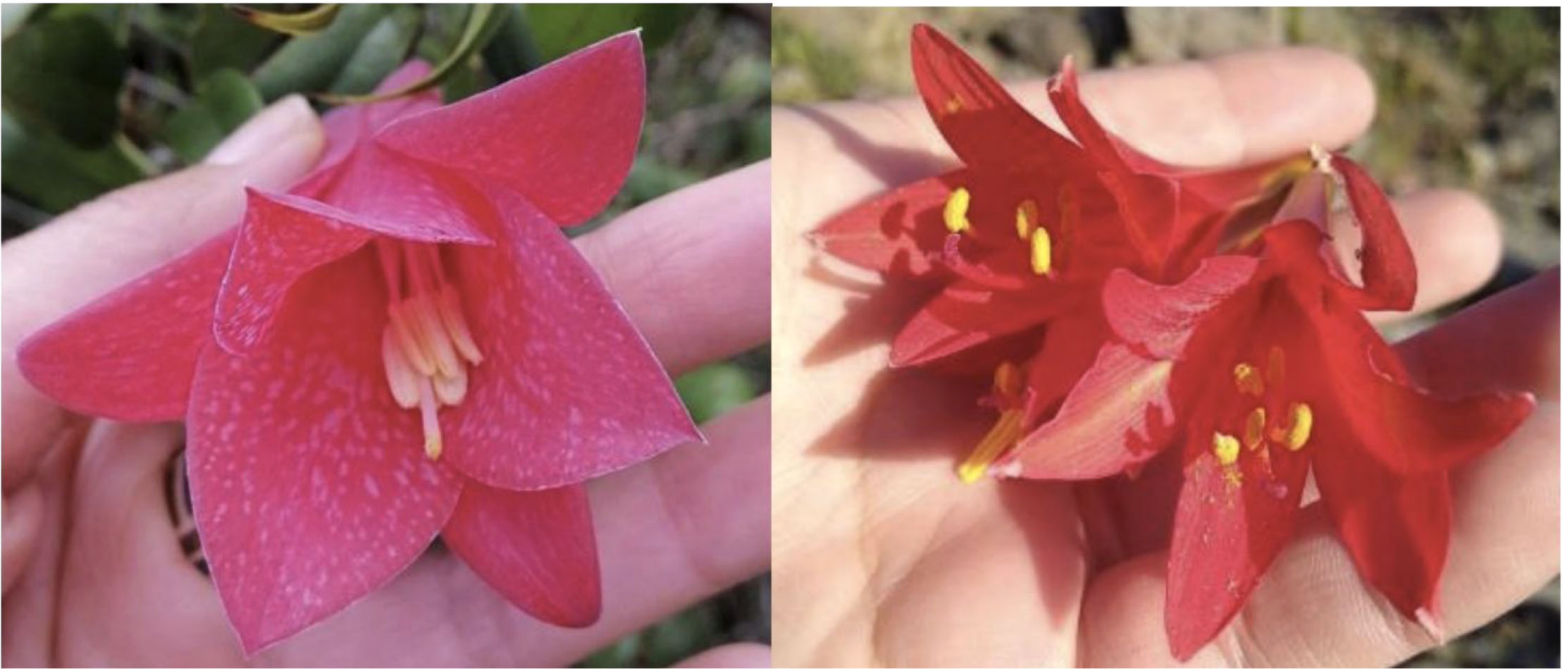
Comparison of similarities between Copihue and Añañuca.

Our selection of flora species was carefully chosen to represent a diverse range of regions in Chile see **Figure 2** while also presenting specific shared traits to add complexity to the classification task. One notable common characteristic among these selected species is the presence of yellow or orange pollen stamens, accompanied by pointed petals rather than rounded ones. The Copihue, Ana˜ nuca,˜ and Chilco classes present a unique challenge in the classification process due to their vibrant red coloration and the potential for their shapes to appear remarkably similar from different angles. These characteristics add complexity to the task of accurately distinguishing and classifying these species (refer to **Figure 3** for visual reference).

Finally, the dataset was divided into two distinct sets: the training set, which comprised 80% of the data, and the testing set, which encompassed the remaining 20%. The decision regarding this split was made based on a careful consideration of various factors, such as the size of the dataset, the desired balance between training and testing data, and the need to ensure a representative sample for evaluating the performance of the classification models (Mohanty et al., 2016).

### 3.3 Data training

During this phase, we initiated the training process by utilizing the ImageNet dataset (Deng et al., 2009), which consists of 1.2 million images distributed across 1000 categories. This served as a starting point to initialize the weights of our convolutional neural networks (CNNs) before fine-tuning them with our specific dataset of native Chilean flora.

To accomplish this, we employed Transfer Learning, a technique that allows the transfer of knowledge from one or more domains to a different domain with a distinct task. In our case, we fine-tuned the pre-trained models on our native Chilean flora dataset. This involved replacing the pre-trained output layer with a new layer that matched the number of classes in our dataset. Consequently, the last three layers of the pre-trained model, which included a fully-connected layer, a softmax layer, and a classification output layer, were substituted.

By utilizing pre-trained CNN models, we benefited from faster and more efficient training compared to starting with randomly initialized weights. Furthermore, pre-trained models exhibited lower training error rates in comparison to artificial neural networks (ANNs) that were not pre-trained. We thoroughly assessed the performance of various CNN architectures in addressing the classification task for native Chilean flora (Zizka et al., 2009).

**Figure 4:**
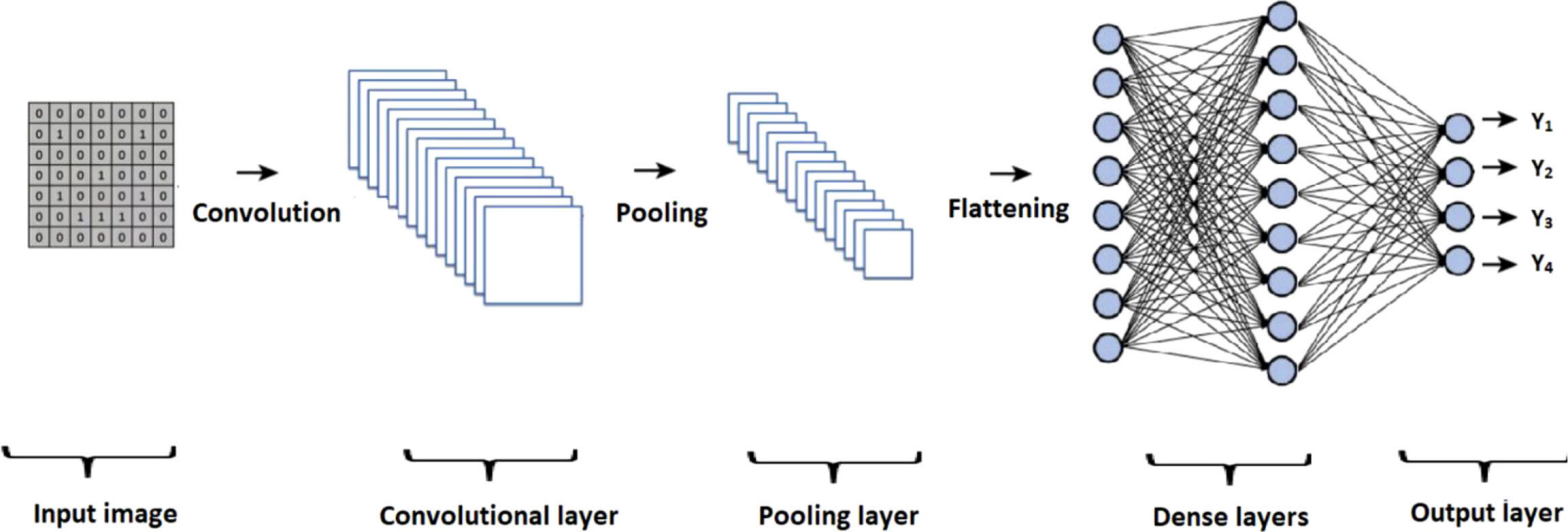
Representation of the architecture of a convolutional neural network (CNN).

CNN architectures are typically composed of specific elements that vary across different models. **Figure 4** presents an overview of the general structure of a CNN, highlighting key components such as the input layer, convolutional layer, pooling layer, and flattening process. The output of the flattening process is then passed through a series of dense layers, culminating in the final output layer.

Therefore, the characteristics of the architectures used are described in the **Table 3**. It is important to highlight that (CNN).

**Table 3:** Summary of the utilized architectures.

Network	Depth	Parameters (Millions)	Image Input Size
VGG19	19	143	224 x 224
ResNET152	152	60	224 x 224
InceptionV3	48	23.9	299 x 299
MobileNetV2	53	3	224 x 224

The InceptionV3 model utilizes a convolutional neural network (CNN) architecture that requires a larger input image size compared to other CNN models, specifically 299x299 pixels. This distinctive image size for InceptionV3 is specifically optimized for the tasks and datasets on which it was trained. It represents a careful balance between maximizing performance on those specific tasks and efficiently managing computational resources.

The choice of a larger input image size, such as 299x299, in InceptionV3 offers several advantages. Firstly, it allows the model to capture more fine-grained details and intricate features within the input images, potentially enhancing its ability to recognize complex patterns. Secondly, the larger image size enables the network to effectively handle a wider range of object scales, accommodating both small and large objects within the same image.

It is essential to note that the specific image size of 299x299 for InceptionV3 is a deliberate design decision based on empirical evaluations and experimentation conducted during its development. This optimization aims to ensure that InceptionV3 performs optimally for the given tasks and datasets it was trained on, providing a balance between accuracy and computational efficiency.

In order to ensure a fair comparison between the experiments, we made an effort to standardize the hyperparameters across all the experiments. The specific hyperparameters used in our experiments are detailed in **Table 4**. The inclusion of these hyperparameters was essential for optimizing the performance of the deep learning models.

**Table 4:** Hyper-parameters of the experiments.

Hyper-Parameters	Value
Optimization algorithm	SGDM
Momentum	0.9000
Initial learning rate	1*x*10^−^3
L2 Regularization	1*x*10^−^4
Epochs	30
Batch size	32

Stochastic gradient descent with momentum (SGDM).

Hyperparameters play a crucial role in controlling different aspects of the training process, including the learning rate, momentum, batch size, and others. By carefully tuning these hyperparameters, our objective was to find the optimal configuration that would facilitate better convergence and improved accuracy of the models.

Standardizing the hyperparameters allowed us to establish a consistent framework for evaluating and comparing the performance of the different CNN architectures. It also ensured that any observed differences in performance were primarily attributed to the architectural variations rather than the hyperparameter settings.

We believe that by employing standardized hyperparameters, we have fostered a more reliable and meaningful comparison between the models, enabling us to draw robust conclusions regarding their relative performance in classifying the images of native Chilean flora.

Deep learning (DL) has revolutionized many research areas. Among optimization algorithms, Stochastic Gradient Descent with Momemtum (SGDM) has emerged as the most widely used due to its balance between accuracy and efficiency (Kleinberg et al., 2018). SGDM is simple and effective, but requires careful tuning of hyperparameters, particularly the initial learning rate, which determines the rate at which weights are adjusted to obtain a local or global minimum of the loss function. Momentum is used to accelerate SGDM in the appropriate direction and reduce oscillations (Ruder, 2016). Regularization is also important to prevent overfitting, with L2 Regularization being the most common type. Combined with SGDM, it results in weight decay, in which the weights are scaled by a factor slightly smaller than one at each update (Van Laarhoven, 2017). To train our models, we used 30 epochs, based on the findings of Mohanty et al. (2016), who reported consistently converging results after the first learning rate decrease. Finally, all CNNs were trained using a batch size of 32.

Training these CNN architectures is extremely computationally intensive. Therefore, all the experiments are carried out on a workstation, presenting the details summarized in **Table 5**.

**Table 5:** Hyper-parameters of the experiments.

Hardware and Software	Characteristics
Memory	16 Gb
Processor	Intel Core i7-7700 CPU @ 3.60 GHz
Graphics	GeForce GTX 1070 X 8 Gb
Operating Systema	Windows 10, 64 bits

Stochastic gradient descent with momentum (SGDM).

### 3.4 Evaluation

The proposed method’s performance is evaluated by comparing pre-trained models using different metrics. The quality of learning algorithms is commonly assessed by how well they perform on test data (Japkowicz and Shah, 2011). One of the metrics used is the Receiver Operating Characteristic Curve (ROC), which is also known as the Area Under the Curve (AUC). The AUC is a widely used performance measure for supervised classification tasks, and it is based on the relationship between sensitivity and specificity (Hanley and McNeil, 1982).

In this work, we used a generalized version of AUC for multiple classes, as defined by Hand and Till (2001). This function calculates the multiclass AUC by taking the mean of several AUC values 1. To use this function, a data frame is passed as a predictor, and the columns must be named according to the levels of the response.


(1) $$AUC=\frac{1}{C(C-1)}\sum_{i=1}^{C}\sum_{j\neq i}^{C}AUC_{ij}$$

where *C* is the number of class in the multiclass problem, *AUC_i_j* presents the binary AUC between *i* and class *j*.

Sensitivity or recall corresponds to the accuracy of positive examples and indicates how many positive class examples were correctly labeled. This can be calculated using Equation 2, where TP represents the true positives, which are the number of positive instances correctly identified, and FN represents the false negatives, which are the number of positive cases as negative.


(2) Sensitivity(Recall)=TPTP+FN

Specificity is a measure of the conditional probability of true negatives given a secondary class, which approximates the probability of the negative label being true. It can be calculated using Equation 3, where TN represents the number of true negatives, i.e., the negative cases that are correctly classified as negative, and FP represents the number of false positives, i.e., the negative instances that are incorrectly classified as positive cases.


(3) $$Specificity=\frac{TN}{TN+FN}$$

To evaluate the overall classification performance, accuracy is the most commonly used metric. During the evaluation stage, accuracy was calculated every 20 iterations. This metric calculates the percentage of samples that are correctly classified, and it is represented by Equation 4:


(4) $$Accuracy=\frac{TP+TN}{TP+TN+FP+FN}$$

Precision is an important metric that evaluates the correctness of a model by measuring the number of true positives divided by the sum of true positives and false positives. In other words, precision measures how many of the predicted positive cases are actually positive, and it assesses the predictive power of the algorithm. The precision score is calculated using Equation 5


(5) $$Precision=\frac{TP}{TP+FP}$$

The F-score is a metric that combines precision and recall, and is defined as the harmonic mean of the two, as shown in Equation 6. It is a measure that focuses on the analysis of the positive class, and a high value of this metric indicates that the model performs better on the positive class.


(6) $$F-score=2*\frac{Precision*Recall}{Precision+Recall}$$

Finally, the data was separated into two sets, containing 80% of the data in the training set and the remaining 20% in the testing set. The choice of the split is based on (Mohanty et al., 2016).

### 3.5 Results

In this study, we evaluated the performance of state-of-theart pre-trained models for the classification of Native Chilean flora. The main objective of this research was to compare the CNN models and assess their accuracy, precision, sensitivity, specificity, F-Score, and AUC through fine-tuning. The results of this evaluation are presented in **Table 6**.

**Table 6:** Performance measures (%) for every pre-trained model.

Measures	VGG19	ResNet152	InceptionV3	MobileNetV2
Accuracy	90.29	95.87	94.98	92.45
Precision	90.81	95.73	95.01	92.74
Sensitivity	90.48	95.23	94.38	92.68
Specificity	91.02	95.87	94.85	93.01
F-Score	90.77	95.85	94.62	93.14
AUC	90.81	96.02	94.82	92.89
Time (milliseconds)	182.12	342.12	652.3	150.23

All models demonstrated similar and statistically significant performance. In terms of AUC, VGG19 and MobileNetV2 yielded the lowest results at 90.81% and 92.89%, respectively, followed by InceptionV3 with 94.82%. The highest AUC result was achieved by ResNet152 with 96.02%, indicating excellent classification. Conversely, VGG19 exhibited the lowest precision metric result at 90.81%, with MobileNetV2 and InceptionV3 following at 92.73% and 95.01%, respectively.

The highest precision was achieved again by ResNet152 at 95.73%. In measures of sensitivity, specificity, and F-score, VGG19 showed poor performance at 90.48%, 91.07%, and 90.77%, respectively. In contrast, ResNet152 had the highest percentage in all previous metrics at 95.23%, 95.87%, and 95.85%, respectively. While all models had statistically significant performance, ResNet152 achieved the highest percentage. Additionally, considering the processing time required by each convolutional neural network (CNN) for the classification task, MobileNetv2 exhibited the best performance with the shortest processing time. This indicates a higher level of efficiency compared to the other CNN architectures. It is worth noting that although MobileNetv2 showed a slight disadvantage in terms of measurement statistics, the difference was not significant when compared to the results obtained by other CNNs, such as InceptionV3, which had the longest processing time. One possible explanation for this observation is that InceptionV3 has a deeper and more complex architecture compared to the other models. Deeper and more complex architectures typically incur a higher computational load, resulting in longer inference times. Thus, it could be considered to accept a minimal decrease in precision in exchange for improved processing efficiency.

**Figure 5:**
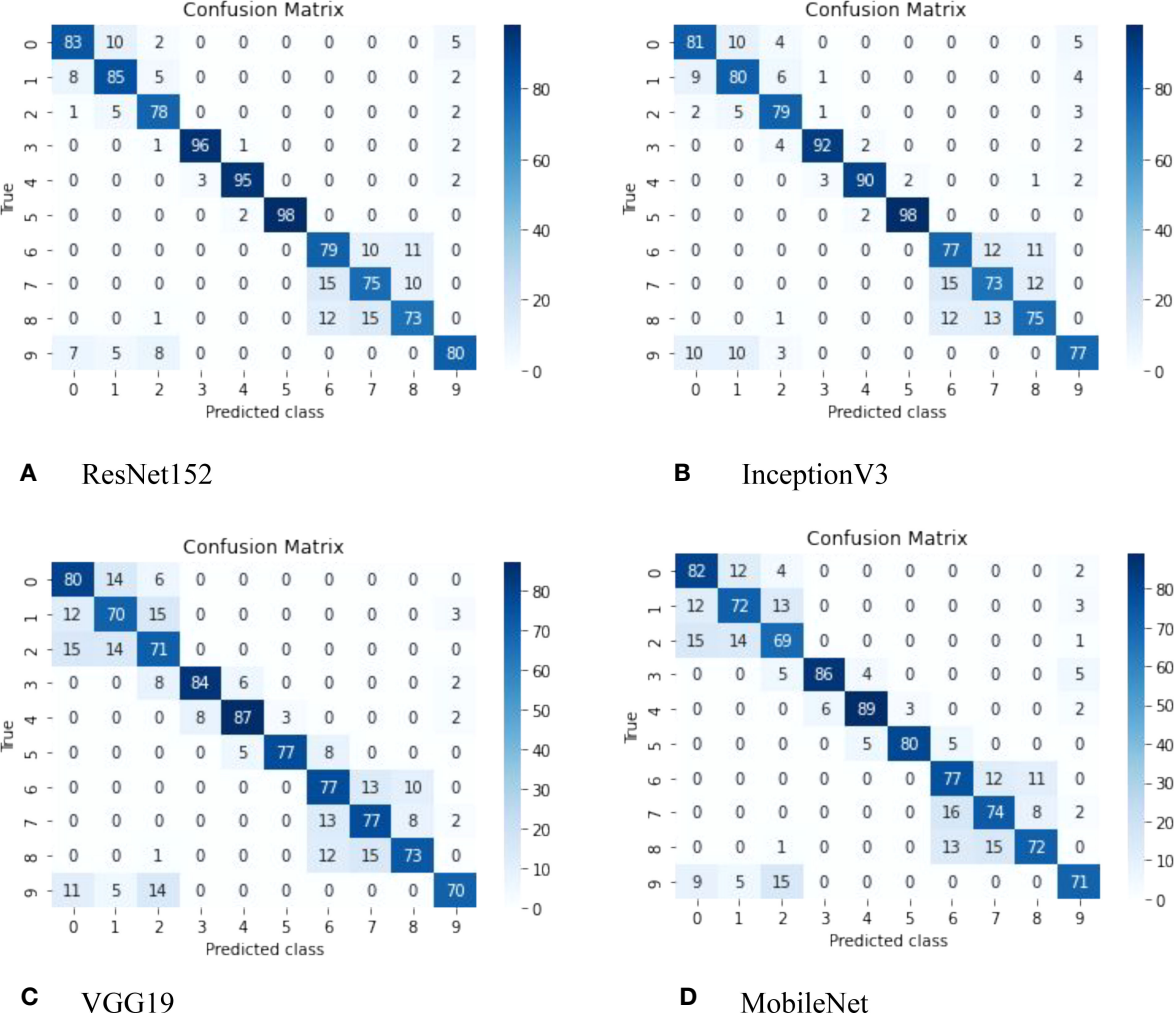
Confusion matrix derived from the ResNet152 model, featuring the following classes: (1) Copihue; (2) Chilco; (3) Añañuca de Fuego; (4) Azulillo; (5) Chagual; (6) Maqui; (7) Lingue; (8) Canelo; (9) Quila; and (10) Notro.

Additionally, **Figure 5** presents the confusion matrix, which visually represents the performance of the classifiers and highlights the classes distinguished by all models used in this study. Each row corresponds to the predicted class, while each column corresponds to the true class. The cells on the diagonal represent correctly classified observations, while the off-diagonal cells indicate misclassifications.

The ResNet152 model has demonstrated superior performance in classifying native Chilean flora compared to the VGG19, InceptionV3, and MobileNet models. This can be attributed to its deep architecture, which enables it to effectively capture complex features and fine details in images.

The ResNet152 architecture utilizes a deep neural network structure with residual layers, allowing it to learn more intricate representations of visual characteristics in plants. This enhanced representation capability enables the ResNet152 model to accurately capture and distinguish the subtle variations and differences among species of native Chilean flora, resulting in higher classification accuracy compared to VGG19, InceptionV3, and MobileNet.

On the other hand, MobileNet stands out for its computational efficiency and processing speed. It achieves this through the use of lighter convolution operations and parameter reduction techniques, resulting in a more lightweight and faster architecture. Although MobileNet may have slightly lower accuracy compared to ResNet152, its processing speed is significantly faster.

The choice between ResNet152 and MobileNet depends on the specific requirements of the application scenario. If achieving the highest accuracy is of utmost importance, and processing time is of secondary concern, ResNet152 would be the preferred choice due to its superior performance in classifying native Chilean flora. However, if reducing processing time is critical, and a slight decrease in accuracy can be tolerated, MobileNet may be the more suitable option due to its computational efficiency and faster processing speed.

Finally, the ability of the ResNet152 model to accurately or inaccurately predict images of Copihue with Chilco and Canelo with Lingue can be attributed to several factors. The following are some possible explanations:(i) Visual similarity: The ResNet152 model has been trained to recognize and distinguish specific visual features of different flora classes. However, it is possible that images of Copihue and Chilco, as well as those of Canelo and Lingue, share similar visual characteristics. These similarities can lead to confusion in the model, resulting in both correct and incorrect predictions. (ii) Intraspecific variability: Within the same species, such as Copihue and Chilco, or Canelo and Lingue, there may be variations in the appearance and characteristics of individual plants. These variations can pose challenges for precise classification, as the model may encounter examples that exhibit atypical or unusual features within the species. In some cases, the model may adapt correctly to these variations and make accurate predictions, while in other cases, it may become confused and make erroneous predictions. (iii) Quality and diversity of the training dataset: The performance of the ResNet152 model heavily relies on the quality and diversity of the training dataset. If the dataset contains a wide variety of images of Copihue, Chilco, Canelo, and Lingue, capturing different variations and characteristics of each species, it is more likely that the model can make accurate predictions. However, if the dataset is limited in terms of species representativeness or does not adequately cover the intraspecific variability, the model may struggle to make precise predictions in all cases.

**Figure 6:**
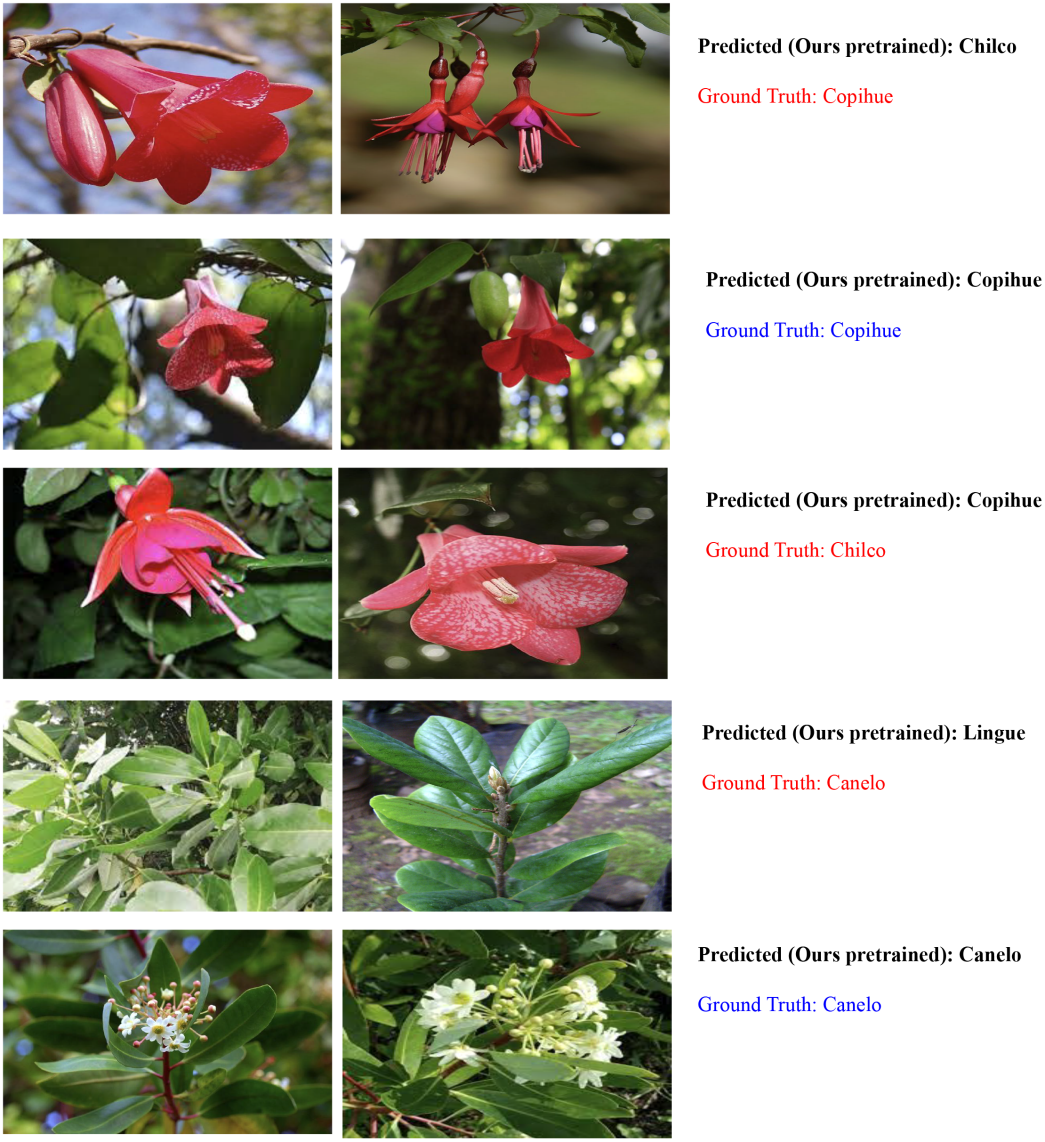
Examples of correct and incorrect predictions on our dataset based on ResNet152.

It is important to note that the performance of the model can be improved through additional techniques such as fine-tuning and optimization of hyperparameters, collecting more representative training data, and including images that encompass a greater variety of species characteristics. These approaches can help reduce prediction errors and enhance the model’s ability to accurately distinguish between Copihue and Chilco, as well as between Canelo and Lingue see **Figure 6**.

### 3.6 Conclusions

In conclusion, this study focused on the creation of a dataset consisting of images of native Chilean flora and the subsequent comparative analysis of different convolutional neural network (CNN) models. The dataset aimed to provide a comprehensive representation of the diverse flora found in Chile, capturing the variations and characteristics of different species.

Through the comparative study, we evaluated the performance of four CNN models: ResNet152, VGG19, InceptionV3, and MobileNet. Our findings indicate that the ResNet152 model exhibited superior performance in classifying native Chilean flora compared to the other models. This can be attributed to its deep architecture, which enabled the model to capture complex features and fine details in images more effectively. The ResNet152 model’s ability to accurately distinguish between species contributed to its higher classification accuracy.

However, it is worth noting that the MobileNet model showcased exceptional computational efficiency and processing speed. While it may have slightly lower accuracy compared to ResNet152, MobileNet’s faster processing speed makes it a suitable choice for scenarios where reducing processing time is crucial and a slight compromise in accuracy can be tolerated.

The study highlighted the importance of the quality and diversity of the training dataset in achieving accurate predictions. Additionally, factors such as visual similarity and intraspecific variability within species were identified as potential challenges in classification tasks.

Overall, this study provides valuable insights into the classification of native Chilean flora using CNN models. The findings can contribute to the development of more accurate and efficient systems for flora recognition and classification, with potential applications in biodiversity conservation, ecological research, and environmental monitoring. Further research can explore advanced techniques to enhance the performance of CNN models and expand the dataset to encompass a broader range of native plant species.

## Data availability statement

The original contributions presented in the study are included in the article/supplementary material. Further inquiries can be directed to the corresponding author.

## Author contributions

CF-F: Conceived and designed the study, performed data collection and analysis, and contributed to the writing of the paper, provided expertise on the subject matter, reviewed and provided feedback on the research methodology and findings, and contributed to the overall manuscript development. PS-M: Assisted with data analysis and interpretation, provided critical feedback on the manuscript. All authors contributed to the article and approved the submitted version.
